# Multivariable and Bayesian Network Analysis of Outcome Predictors in Acute Aneurysmal Subarachnoid Hemorrhage: Review of a Pure Surgical Series in the Post-International Subarachnoid Aneurysm Trial Era

**DOI:** 10.1093/ons/opx163

**Published:** 2017-07-31

**Authors:** Zsolt Zador, Wendy Huang, Matthew Sperrin, Michael T Lawton

**Affiliations:** 1 Department of Neurosurgery, Salford Royal NHS Foundation Trust, Salford, United Kingdom; 2 Institute of Cardiovascular Sciences, Centre for Vascular and Stroke Research, University of Manchester, Manchester, United Kingdom; 3 Department of Neurosurgery, University of California, San Francisco, San Francisco, California; 4 Farr Institute, Faculty of Biology, Medicine and Health, University of Manchester, Manchester Academic Health Science Centre

**Keywords:** Subarachnoid hemorrhage, Outcome predictors, Machine learning, Bayesian networks

## Abstract

**BACKGROUND:**

Following the International Subarachnoid Aneurysm Trial (ISAT), evolving treatment modalities for acute aneurysmal subarachnoid hemorrhage (aSAH) has changed the case mix of patients undergoing urgent surgical clipping.

**OBJECTIVE:**

To update our knowledge on outcome predictors by analyzing admission parameters in a pure surgical series using variable importance ranking and machine learning.

**METHODS:**

We reviewed a single surgeon's case series of 226 patients suffering from aSAH treated with urgent surgical clipping. Predictions were made using logistic regression models, and predictive performance was assessed using areas under the receiver operating curve (AUC). We established variable importance ranking using partial Nagelkerke R^2^ scores. Probabilistic associations between variables were depicted using Bayesian networks, a method of machine learning.

**RESULTS:**

Importance ranking showed that World Federation of Neurosurgical Societies (WFNS) grade and age were the most influential outcome prognosticators. Inclusion of only these 2 predictors was sufficient to maintain model performance compared to when all variables were considered (AUC = 0.8222, 95% confidence interval (CI): 0.7646-0.88 vs 0.8218, 95% CI: 0.7616-0.8821, respectively, DeLong's *P* = .992). Bayesian networks showed that age and WFNS grade were associated with several variables such as laboratory results and cardiorespiratory parameters.

**CONCLUSION:**

Our study is the first to report early outcomes and formal predictor importance ranking following aSAH in a post-ISAT surgical case series. Models showed good predictive power with fewer relevant predictors than in similar size series. Bayesian networks proved to be a powerful tool in visualizing the widespread association of the 2 key predictors with admission variables, explaining their importance and demonstrating the potential for hypothesis generation.

**ABBREVIATIONS**
AUC: area under the curve
CI: confidence interval
DAG: directed acyclic graphs
GOS: Glasgow Outcome Scale
INR: international normalized ratio
ISAT: International Subarachnoid Aneurysm Trial
WFNS: World Federation of Neurosurgical Societies

Treatment of acute subarachnoid hemorrhage (aSAH) has undergone substantial evolution over the past 3 decades.^1^ Surgical clipping remains an essential treatment modality. With the benefits of endovascular coiling formalized by class 1 evidence, the clinical caseload has become divided, with the more challenging cases often being diverted toward open clipping.^2-4^ In their single-center case series, Gnanalingham et al^3^ noted an increase in the percentage of coiled aneurysms from 35% to 67% over a period of 27 mo, centered around the publication of the International Subarachnoid Aneurysm Trial (ISAT) in October 2002, with a parallel decrease in clippings. Sanai et al^4^ assessed the case mix of aneurysms undergoing clipping over an 11-yr period (1997-2008) in terms of complexity features, such as aneurysm morphology and the advanced surgical techniques required to successfully treat the aneurysm. They found that almost double the number of posterior communicating artery aneurysms had multiple complexities (25% vs 15%) in the second half (post-ISAT period) of the case series. Another relevant factor in the changing treatment of acute subarachnoid hemorrhage is perioperative management, particularly the evolution of support from intensive care, which translates into the 17% reduction in case fatality of aSAH. Given these recent changes in the clinical landscape, we conducted a pilot analysis of early outcome predictors in a pure surgical case series using formal importance ranking in multivariable regression models and machine learning techniques.

Multivariable regression has been widely used for outcome prediction in clinical data sets.^6,7^ This technique is capable of determining the prognostic value of each variable and adjusts for the confounding of other variables. It has yielded a number of predictive models in the field of neurotrauma and has provided a practical option to refine clinical decision making, inform patient/relative expectations, and help in the designing of clinical trials.^7^ Modern statistical techniques in biomedical sciences allow further insight into outcome prediction and data structure. Partial Nagelkerke R^2^ score allows formal ranking of outcome predictors based on their contribution to how well the prediction model fits the input data.^7^ Furthermore, the performance of the predictive model can be assessed based on the area under the receiver operating curve (AUC). Machine learning methods such as Bayesian networks allow a picturesque view of probabilistic associations of each variable, therefore informing on the influence they have on one another.^8-10^ Using these techniques, our aim was to identify the most influential outcome predictors in aSAH and to explain their importance by analyzing their associations with the remaining variables.

## METHODS

### Patient Database

The study was approved by the Institutional Review Board at our center. For the purposes of this pilot study, we retrospectively reviewed the neurovascular database at our department. We included all patients presenting with aSAH who subsequently underwent urgent surgical clipping between 2011 and 2015. This search yielded a total of 246 consecutive patients. Patients with incomplete assessment data were excluded (18 patients). To create a predictive model of early clinical outcome, we considered Glasgow Outcome Scale (GOS)^11^ at the time of discharge from the center. We dichotomized moderate to low disability as a good outcome, whereas severe disability, persistent vegetative state, and death were classed as a poor outcome, as described previously.^12^ Clinical variables included demographics, cardiorespiratory observations, admission laboratory values, World Federation of Neurosurgical Societies (WFNS) grade, Fisher grade, hydrocephalus, and seizures on presentation (summarized in Table 1). Laboratory results were dichotomized as guided by standard reference values.^13^ We dichotomized clinical outcome as favorable (GOS 4 and 5: moderate to low disability, respectively) and unfavorable (GOS 3, 2, and 1: severe disability, persistent vegetative state, and death, respectively). We carefully considered the potential problem of losing valuable clinical information by collapsing a multilevel clinical scale (the GOS) into a binary measure. We gave thought to circumventing this problem by using methods such as a sliding dichotomy and proportional odds model. These two methods were suggested as having distinct advantages over a fixed dichotomy, such as a reduction in sample size without loss of statistical power^14^ in traumatic brain injury data sets. On the other hand, Ilodigwe et al^15^ found no benefit in applying these methods to a subarachnoid hemorrhage data set compared to the conventional fixed dichotomy. Our study uses variable importance ranking and Bayesian networks, which technically limited our interface to dichotomized outcomes. Previous studies have also favored fixed dichotomy, and continuing with this technique allowed for better interpretation of our results. Furthermore, dichotomized outcomes lend themselves to well-established evaluation methods such as receiver operating curve. Although the concept of alternative methods was appealing, given the reasons above, we chose to stay with a fixed dichotomy.

**TABLE 1.** Summary of Continuous and Categorical Variables Considered in Our Analysis

Admission variable	Frequency %/average ± stdev	Label on DAG (Figure 2)
GOS 4 and 5	30.08	outcome
Patient age	56.81 ± 14.71	age
Male gender	27.43	gender
Oxygen support (>2 L)	45.13	oxygen
Systolic blood pressure >160 mm Hg	26.1	syshigh
Systolic blood pressure <90 mm Hg	2.21	syslow
On antocoagulation	10.17	anticoag
INR > 1.3	8.84	inr
Platelets < 150 × 10^3^ cells/μL	7.96	ptls
HbG < 13.5 g/dL (males) HbG < 12 g/dL (females)	32.3	HbG
WBC > 10.5 × 10^3^ cells/μL	71.23	wbc
Glucose > 150 mg/dL	58.84	glucose
Early seizures	8.85	seizures
Hydrocephalus	69.8	hydroceph
WFNS grade		wfns
1	43.81	
2	7.08	
3	4.42	
4	34.96	
5	9.73	
Fisher grade		fisher
1	0.88	
2	7.52	
3	25.66	
4	65.93	

### Predictive Models

We applied logistic regression models for predictions and to assess variable correlation with clinical outcome. For model selection, we used Akaike information criterion with backward elimination to optimize the balance of model complexity against goodness of fit.^16^ Predictions were undertaken with 5-fold cross validation to avoid overfitting.^10,17^ This method has been found superior in terms of discriminatory ability, calibration, and overall accuracy to the split-sample method by the comparative study of Steyerberg et al.^18^ Predictive performance of the different models described below was assessed by computing the AUC and compared using DeLong's test.^19^

### Variable Importance Ranking

We used the Nagelkerke R^2^ value, a measure for goodness of fit, to rank variable importance. Nagelkerke R^2^ numerically expresses the percentage of variability attributed to a predictor. The ranking of variables was extracted from the drop in the Nagelkerke R^2^ value that occurred in response to excluding variables of interest from the model. We used this ranking to identify the most influential variables and include them to create a more simplistic model with fewer variables. There are recognized limitations for “pseudo” R^2^ methods: (1) they can be argued to give artificially high R^2^ scores that may suggest the model fits better than it really does, and (2) there are a variety of “pseudo” R^2^ measures to choose from, each of which interprets the model differently and therefore gives different results. In our study, we used the same modality of R^2^ value to assess the change in model fit rather than focus on the numeric value, a technique that has been applied in multiple papers.^7,10^

### Assessing Probabilistic Associations Using Bayesian Networks

Bayesian networks depict probabilistic relationships between variables^10,20^ using directed acyclic graphs (DAG). The DAG comprises nodes, representing clinical variables, and edges that connect nodes, indicating the conditional dependence between them. To establish which network structure best describes the probabilistic relationships between the variables, we used the hill-climbing algorithm^10,21^ to search the possible networks.

### Statistical Software

All statistical analysis and modeling was carried out in R,^22^ an open-source software environment for statistical programing and graphics (https://www.r-project.org/). Receiver operating curve analysis was carried using the “pROC” package.^23^ Threshold optimization was performed using packages “pROC” and “SDMTools.”^24^ AUCs were compared using DeLong's test.^19^ Nagelkerke R^2^ was implemented using the “fmsb” package.^25,26^ The “bnlearn”^27^ package was used for Bayesian network analysis.

## RESULTS

### Patient Characteristics

Patient characteristics and admission variables are summarized in Table 1. Mean follow-up was 19.08 ± 11.44 d, and average time to clipping was 2.93 ± 3.22 d (average ± standard deviation).

### Multivariable Analysis and Variable Importance Ranking

**FIGURE 1.**
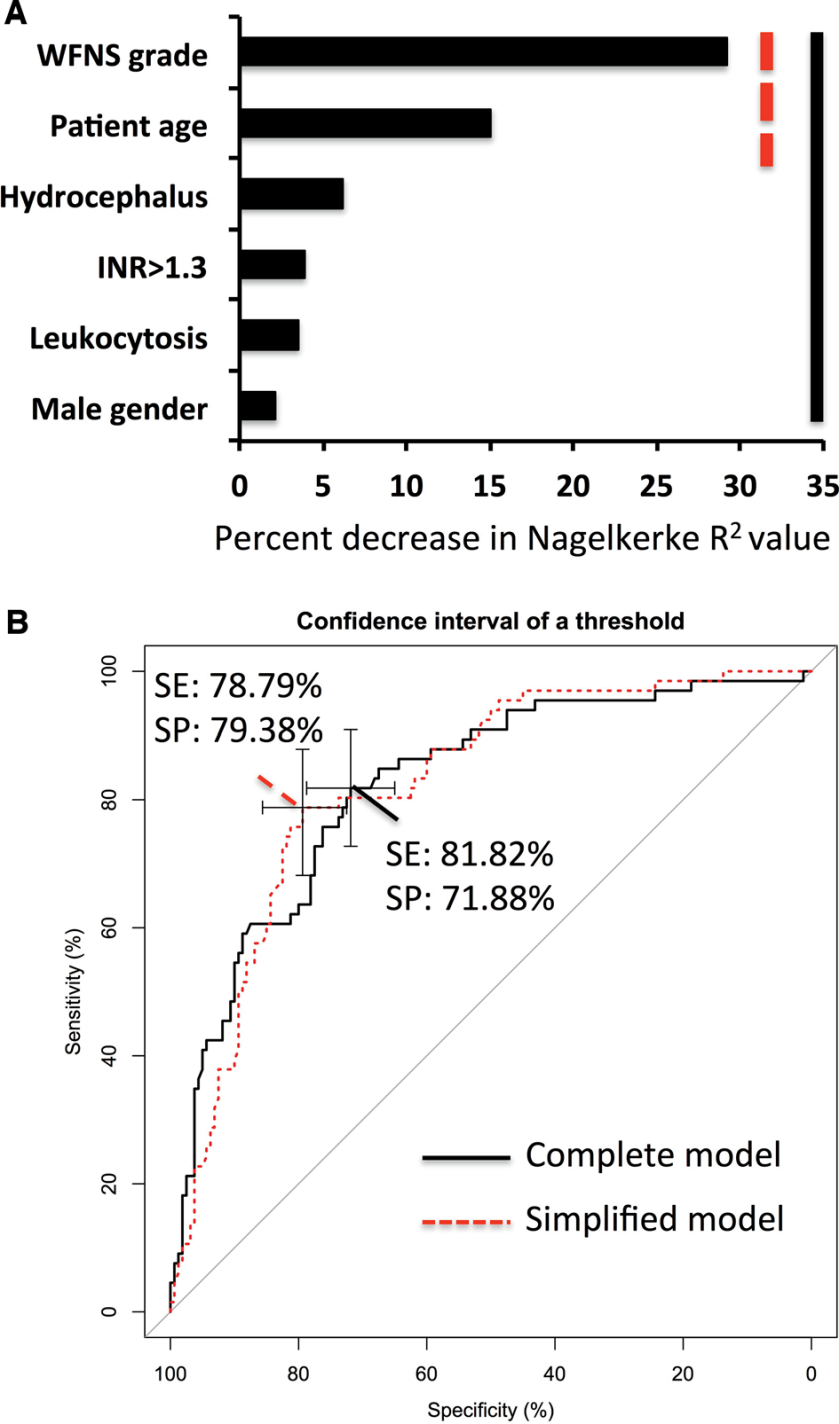
**A**, Importance ranking of variables using partial Nagelkerke R^2^ scores. Variables incorporated into the complete model are highlighted by the black bar, and those used in the simplified model are indicated by the interrupted red bar. **B**, Receiver operating characteristic curve for complete (continuous line) and simplified (red interrupted) logistic regression models. Threshold was selected to achieve optimal sensitivity and specificity.

Backward elimination selected WFNS grade, age, hydrocephalus, international normalized ratio (INR) equal or greater than 1.3, white blood cell count over 10.5 × 10^3^ cells/μL (leukocytosis), and male gender to be included in the logistic regression model. Analysis demonstrated that lower WFNS grade, younger age, and absence of acute hydrocephalus on admission were significantly correlated with good clinical outcome (GOS 4 and 5). To better illustrate the odds ratio associated with these variables, a dichotomized model was also created as summarized in Table 2. Variable importance ranking based on partial Nagelkerke R^2^ values is summarized in Figure 1A, with WFNS grade and age shown as the most influential variables.

**TABLE 2.** Results Summary From Optimized Regression Models. Patient Age and WFNS Grade were Considered as Continuous Then as Dichotomized Variables

Variable	Coefficient	Standard error	Odds ratio (favorable outcome)	Confidence Interval (97.5%)	*P*-value
Continuous					
Intercept	3.19	0.82	N/A	N/A	<.001
Patient age	−0.047	0.013	0.95	0.93-0.98	<.001
Male gender	0.57	0.39	1.77	0.82-3.82	.14
INR > 1.3	−1.82	1.13	0.16	0.008-1.01	.11
WBC > 10.5 cells/μL	0.76	0.42	2.13	0.96-4.96	.07
WFNS grade	−0.68	0.14	0.51	0.38-0.66	<.001
Hydrocephalus	−0.95	0.39	0.39	0.18-0.82	<.001
Dichotomized					
Intercept	−3.67	0.81	N/A	N/A	<.001
Age below 65	2.12	0.57	8.35	2.99-29.93	<.001
Male gender	0.44	0.39	1.55	0.72-3.33	.26
INR > 1.3	−1.66	1.18	0.19	0.009-1.34	.16
WBC > 10.5 cells/μL	0.58	0.41	1.78	0.81-4.06	.16
WFNS grade	1.98	0.43	7.23	3.22-17.52	<.001
Hydrocephalus	−1.05	0.38	0.35	0.16-0.75	<.01

### Prediction of Clinical Outcome

For predictions, we used the model built with backward elimination as described previously. AUC for this predictive model was 0.8218 (95% confidence interval (CI): 0.7616-0.8821), translating into 81.82% median sensitivity and 71.88% median specificity (Figure 1B).

### Model Selection Based on Variable Importance Ranking

We tested which variables were essential to maintain the accuracy of the predictive model. Inclusion of the 2 highest ranking variables WFNS grade and age alone gave an AUC of 0.8223 (95% CI: 0.7646-0.88) with 78.79% sensitivity and 79.38% specificity (Figure 1B). This simplified model yielded equally accurate predictions as the complete model described earlier (DeLong's test *P* = .992).

### Probabilistic Associations Between Key Outcome Predictors and Remaining Variables

**FIGURE 2.**
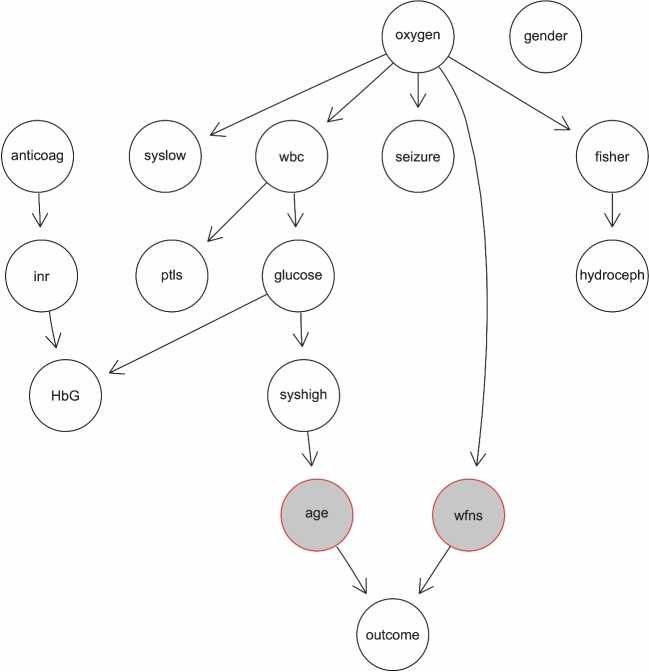
DAG depicting Bayesian networks with probabilistic associations between key predictors (highlighted in gray) and remaining variables. Note the widespread influence of oxygen requirements and the associations of laboratory/cardiovascular parameters with age. See Table 1 for abbreviations.

In the Bayesian network analysis, the influence of age and WFNS grade on outcome was prefixed using the “whitelist” argument in the “bnlearn” package to demonstrate their key predictor role in the simplified model. The probabilistic relationships outside this constraint were explored using the hill-climbing search. The network analysis showed widespread probabilistic associations between the key predictors and the remaining variables (Figure 2). Age was indirectly associated with variables through systolic blood pressure over 160 mm Hg, whereas WFNS grade was associated with oxygen requirements. The need for oxygen support appeared to have further associations with several admission variables besides WFNS grade, such as Fisher grade, early seizures, and systolic blood pressure below 90 mm Hg. Intuitive associations in other parts of the networks were anticoagulation to raised INR influencing low hemoglobin. Furthermore, Fisher grade was associated with the presence of hydrocephalus, as shown by previous results.^28^

## DISCUSSION

Our preliminary report gives an update on outcome predictors for a pure surgical case series in the post-ISAT era of neurovascular surgery. We applied established statistical methods to optimize logistic regression models, which achieved over 75% sensitivity and specificity at predicting a dichotomized clinical outcome. Our study is the first to adopt the methods of variable importance ranking to subarachnoid hemorrhage in a pure surgical series post-ISAT. This concept can help with refocusing our clinical assessment and subsequent decision making. Our model selection was based on variable contribution to predictive performance and yielded a relatively parsimonious model compared to recent studies of comparable sample size.^28-31^ In line with a previous study,^32^ we found age and WFNS grade to be the most important predictors. Our study is the first to demonstrate that inclusion of only these 2 variables was sufficient to predict clinical outcome with equal accuracy to more complex models. Using Bayesian networks, we further demonstrated that these 2 key predictors were influenced by the remaining variables, which may explain why the simplified model has maintained its accuracy. We also found that some of the associations revealed by the Bayesian network paralleled our clinical expectations and also raised new research questions. These latter findings suggested that Bayesian networks are efficient at formalizing clinical intuition and also allow hypothesis generation.

### Multivariable Predictive Models in Subarachnoid Hemorrhage

Predicting surgical outcomes using modern statistical models is of increasing interest due to their ability to inform clinical decision making and patient/relative expectations.^6,7,10^ Recent case series of aSAH have demonstrated several admission variables associated with clinical outcome in a mixed clipping and coiling case series.^29-32^ Variables such as blood transfusion, pyrexia, hyperglycemia, hypotension with vasopressor requirements, posterior circulation aneurysm, early onset seizures, hematoma, and ischemic stroke on imaging correlated significantly with poor clinical outcomes in logistic regression models. Although the types of clinical variables analyzed by these studies are not fully consistent, age and WFNS grade appears to be a common predictor of poor outcome. Rosengart et al^32^ amalgamated 4 randomized, double-blind, placebo-controlled trials from the 1990s for analysis of outcome predictors. They demonstrated admission neurological grade as the highest ranking variable followed by patient age based on proportion of explained variance in multivariable logistic regression model. Our study conveys the same findings, suggesting that despite the shift in treatment paradigm, the same clinical features continue to be the main predictors of outcome. Our predictive model achieved similar accuracy compared to more complex models, which included numerous predictors by incorporating only age and WFNS grade (AUC 0.8218 vs 0.8223 in our study). Furthermore, our study focuses on admission variables in a pure surgical case series of a single surgeon. With the current models, we aim to reflect the clinical information available pretreatment, which is meant to inform early decision making. Therefore, later stages of the patient's clinical journey, such as delayed ischemic deficits, pretreatment rebleeds, postprocedure complication, or sepsis are not represented. Analysis of these variables would constitute a separate study, and we have ongoing work with modeling these stages of the patient clinical journey.

### Machine Learning: An Emerging Technique in Biomedical Sciences

Machine learning has the ability to process high-dimensional data sets for complicated tasks such as image recognition, language processing,^33^ or radiological image analysis.^34^ Techniques are evolving rapidly, and their application in clinical neurosciences holds great potential. In the current study, we used Bayesian networks, a more classic example of machine learning, in which probabilistic relationships between clinical variables can be established using an automated algorithm. These relations are then displayed using a DAG, in which “nodes” represent clinical predictors and “edges” between nodes highlight the probabilistic relationships. This technique allows insight into data structure (ie, which variable influence each other), which is a distinct advantage over conventional statistical methods. In a recent study, we used this technique to explore probabilistic relationships between predictors of clinical outcome in traumatic brain injury.^10^ Through an automated search process, we found intuitive associations between clinical variables: age was related to mechanisms of injury, and pupil reactivity was related to mass effect on admission computed tomography scan. Artificial neuronal networks are another example of machine learning, which are quite distinct from Bayesian networks. This technique was inspired by the biological structure of nervous tissue, in particular the way neurons process synaptic input and subsequently communicate with one another. Nodes are equivalents to neurons (rather than the nodes representing variables as in Bayesian networks), and these nodes are connected by edges analogous to synapses between the neurons. The fundamental structure of the neuronal network can be predefined, and the nodes are initially trained with input data to fire only above a given threshold. Further important components are the weight assigned to the edge, representing the influence of the synapse, and a bias, which adjusts the activation threshold of the neuron. With multiple layers of nodes connected to one another (also called a deep neuronal network), this technique can be used to process rather detailed data such as images, handwriting, or even human voices for recognition. In the biomedical field, recent studies have applied artificial neuronal networks to predicting vasospasm in subarachnoid hemorrhage with encouraging results.^35^ One of the most recently developed techniques of machine learning is “deep learning,” which essentially represents a sophisticated neuronal network consisting of several node layers. There have been some striking results in the recent literature using this method. Hassabis's group has reported deep learning to achieve human performance at beating classic Atari 2600 games^36^ and even defeat human professionals at the game of Go.^37^ Translating this method to the medical field carries revolutionary potential. Studies so far have demonstrated deep learning to distinguish between calcification and carcinoma on mammograms with great efficacy.^34^ These techniques, however, require sufficiently complex data for their potential to unfold. Suggested by results from a preliminary study from our group, we found that machine learning gave similar prediction accuracy as logistic regression when applied to traumatic brain injury data sets.^38^ When applied to highly structured data (such as clinical trials databases), machine learning techniques are likely to detect the same patterns as logistics regression yielding similar performance. However, the ability to formalize intuitive clinical associations remains a distinct advantage of machine learning, as demonstrated in the current and previous studies.^10^ One promising trajectory for machine learning and clinical prediction models would be the combination of unstructured imaging data and clinical text from electronic patient records to potentially boost the performance of existing predictive models.

### Model Building and Bayesian Network Analysis of Variable Importance

As discussed above, several clinical features were demonstrated to be important at determining outcome. Our study shows that a limited amount of clinical information in a simplified model was sufficient to predict outcome with accuracy equal to more complex models. Recent studies have described model performance by computing AUC^30,32^; others have used it as an endpoint to examine the contribution of variables to predictive model accuracy.^10,12^ As demonstrated by our results, statistical correlation with outcome does not necessary mean the same variable will be useful at informing predictions. This means that excluding lower ranking variables from the predictive model does not change prediction accuracy. Using Bayesian networks, we showed that the high-ranking predictors of WFNS grade and age hold probabilistic associations with the remaining clinical variables, and therefore their effect is carried on into the predictive model through these probabilistic effects. Bayesian networks allow a visual demonstration of probabilistic influence between variables, therefore representing a convenient technique to test variable influence on the 2 key predictors in our study. Our network analysis showed that admission cardiovascular parameters and blood glucose levels influence outcome through patient age. This may point toward the impact of comorbidities such as hypertension and diabetes on outcome. Another finding was the widespread association of oxygen support with several variables. The explanation for this could be the protocol-driven administration of oxygen to patients with low GCS score (corresponding to high WFNS grade) or seizures. There were further intuitive associations in parts of the network that were more distant from outcome, such as the influence of anticoagulation on high INR and low hemoglobin. Another finding was the influence of Fisher grade on hydrocephalus, an association well documented in the literature.^39,40^ The sensitivity of Bayesian networks to these associations highlights it as a powerful method to formalize clinical intuition and can potentially help hypothesis generation in future studies.

There is robust evidence that modern perioperative care is effective at recognizing and treating important medical/surgical problems mirrored by the improvement in patient mortality;^5^ in particular, the introduction of multidisciplinary neurocritical care translated into substantial improvements in patient outcome in acute aSAH.^1^ Such advances can interpret the relatively low predictive importance of other admission variables such as Fisher grade, early seizures, low hemoglobin, or low platelet count. Furthermore, the high importance and predictive accuracy of age and WFNS grade in contrast to the above factors suggests that our clinical management is successful at reducing the impact of “treatable” risk factors of poor outcome.

### Limitations

Our study only considered early clinical outcomes, which does not capture the substantial progress patients can make during the rest of their clinical course, particularly during their rehabilitation period.^41^ Our predictions are made using a single-center data set, which could result in confounding from characteristics of the local patient population, neurosurgical care setup, and surgical techniques. Validation of our findings using an external data set is therefore desirable.

Although the topic is relevant, neither current nor prior studies offer a complete analysis on treatment trends for aneurysms over the pre- and post-ISAT era. We were also limited by logistics, as the neurointerventional data are managed separately from the vascular neurosurgery database in our department. Such an analysis is beyond the scope of our paper and would constitute a separate study.

## CONCLUSION

We provide the first analysis of early clinical outcomes in a pure surgical case series in the post-ISAT era. Our preliminary study highlights the benefits of insight and hypothesis generation for modern statistical methods in clinical data analysis: (1) identification of influential variables allows the selection of simplified models while maintaining prediction accuracy, and (2) probabilistic associations between variables may propose previously unappreciated correlations, prompting future studies. Although we were limited to a single surgeon experience, our results promote the application of modern statistical methods, including machine learning, to larger, multicenter databases in future studies.

### Disclosures

Zsolt Zador is the recipient of an Academic Clinical Lectureship from the National Institute for Health Research. Matthew Sperrin has received funding from the Medical Research Council (grant number: MR/K006665/1). The authors have no personal, financial, or institutional interest in any of the drugs, materials, or devices described in this article.

## COMMENT

In this retrospective review of 226 acute subarachnoid patients who underwent surgical clipping at a single-institution by a single surgeon, the authors aimed to identify outcome predictors. Multiple clinical variables were evaluated and outcome was measured by the Glasgow Outcome Scale at the time of discharge.

The opportunities to exploit the advantages of machine learning, such as any multi-variable statistics and Bayesian networks for health care research are exciting, though limitations and concerns must be considered. The authors' concluding remarks support the benefit of insight and hypothesis generation but also promote the use of probabilistic analytics to suggest new associations to drive future studies. One may argue that these 2 points may be contradictory as conducting a study in aims to prove results from machine learning networks may innately create bias.

Using Bayesian networks for analysis of outcome predictors begins with probable associations and assumptions using previous data and results in statements yielded from a certain degree of inference. The interpretation therefore must be taken with the understanding of conditionally independent variables outside of its descendants and Markov's blanket. The authors are mixing multiple statistical methods that are considered novel and utilize a chained analysis, which is extremely difficult to evaluate for its ability to deliver results that can be trusted. Cardiovascular parameters and blood glucose levels influencing outcomes through patient age are examples of these inferences and, more simply, are just noted associations.

The article encourages thought-provoking discussion and opens a nuanced way of evaluating and treating these complex patients, which is directly in line with the recent and current health care reforms on value of care.^1^ As elegantly stated by the authors, clinical evaluations post-discharge may significantly change reported patient outcomes. It is unfortunate that it remains unclear how the methods used here actually relate to other publications that use different methods and why the authors believe that using some different statistical methods is superior. It would be interesting to see if there would be any change in networks after new reports of long-term patient outcomes. Improved short-term outcomes may prove to be a predictor of improved long-term outcomes. The application of such associations is key to these types or reports. Prospective randomized-controlled trials evaluating modifiable perioperative risk factors that can affect outcome can yield fruitful results.

**Sanjay Konakondla**

*Danville, Pennsylvania*

**Clemens M. Schirmer**

*Wilkes Barre, Pennsylvania*
